# Juxtacortical Lesions in Multiple Sclerosis: Assessment of Gray Matter Involvement Using Phase Difference-enhanced Imaging (PADRE)

**DOI:** 10.2463/mrms.mp.2015-0099

**Published:** 2016-02-03

**Authors:** Koichiro FUTATSUYA, Shingo KAKEDA, Tetsuya YONEDA, Issei UEDA, Keita WATANABE, Junji MORIYA, Yu MURAKAMI, Satoru IDE, Atsushi OGASAWARA, Norihiro OHNARI, Kazumasa OKADA, Hiroaki ADACHI, Yukunori KOROGI

**Affiliations:** 1 Department of Radiology, University of Occupational and Environmental Health, School of Medicine, 1-1 Iseigaoka, Yahatanishi-ku, Kitakyushu 807-8555, Japan; 2 Department of Medical Physics in Advanced Biomedical Sciences, Kumamoto University; 3 Department of Neurology, University of Occupational and Environmental Health

**Keywords:** multiple sclerosis, phase difference enhanced imaging, juxtacortical lesion, gray matter involvement

## Abstract

**Purpose:**

In multiple sclerosis (MS), a juxtacortical lesion at the border between the gray matter (GM) and subcortical white matter (WM) may often involve the GM. A recently developed, phase-weighted magnetic resonance imaging (MRI) technique “phase difference enhanced imaging (PADRE)” can delineate the GM and WM clearly due to the difference in myelin concentration. We evaluated whether PADRE is useful for the detection of GM involvement in the juxtacortical MS lesions.

**Methods:**

One neuroradiologist reviewed the conventional MRI in 13 MS patients and selected 48 juxtacortical lesions. At the first reading session with the conventional MRI alone (T_2_-weighted imaging, and two-dimensional and three-dimensional fluid-attenuated inversion recovery), two other neuroradiologists classified the lesions into three patterns according to their anatomical locations: (a) subcortical WM lesions involving the subcortical WM alone; (b) intracortical (IC) lesions involving the GM alone; (c) mixed GM/subcortical WM (mixed) lesions involving the both subcortical WM and GM. We defined the subcortical WM as a WM within a distance of 10 mm from inner edge of the GM. For the analyses, we excluded the white matter lesions further than 10 mm from inner edge of the GM. At the second reading session MRI and PADRE were available and the radiologists re-evaluated their prior classification.

**Results:**

At the first reading session, 27 lesions were classified as (a), 1 as (b), and 20 as (c). Therefore, a total of 21 lesions (44%) were judged to involve the GM. At the second reading session, the classification of 15 (31%) lesions changed; all 15 lesions were judged to involve the GM on the PADRE. Interobserver agreement (kappa value) was 0.84 for the first- and 0.95 for the second reading session.

**Conclusion:**

PADRE is useful for detecting GM involvement of the juxtacortical MS lesions.

## Introduction

Multiple sclerosis (MS), an inflammatory demyelinating disease of the central nervous system, usually affects young adults and leads to chronic invalidism. The high sensitivity of magnetic resonance imaging (MRI) facilitates the detection of macroscopic tissue abnormalities in MS patients. Conventional MRI studies such as T_2_-weighted imaging (T_2_WI), fluid-attenuated inversion recovery (FLAIR) imaging, and T_1_-weighted imaging (T_1_WI) with and without gadolinium-based contrast enhancement yield information important for diagnosing MS, for understanding its natural history, and for assessing the efficacy of treatment.

Histopathologically, in patients with MS, a juxtacortical lesion at the border between the gray- and subcortical white matter (GM, WM) often involves the GM.^1–3^ Although the plaque burden within the GM seems to be correlated with neurologic impairment in MS patients, on conventional MRI scans it can be difficult to identify GM involvement. Double inversion recovery (DIR) MRI ^4–6^ and phase imaging using high-field MRI^7,8^ are superior to conventional MRI for depicting MS lesions close to or in the GM. However, the signal-to-noise ratio (SNR) on DIR MRI scans is low, although the patient-specific optimization of three-dimensional (3D)-DIR is now feasible and further improves GM-WM contrast.^9^ Moreover, 7T MRI can only be used in research studies. A new phase-weighted MRI technique, phase difference-enhanced imaging (PADRE), uses the phase difference between the target and surrounding tissue to enhance the contrast of the target tissue.^10,11^ Choosing the appropriate phase differences allows the variation in tissue contrast using single imaged magnetic resonance (MR) data. Some PADRE images yield a level of contrast that could not be realized with earlier phase techniques. Kakeda et al.^10^ and Ide et al.^12^ who reported that various fibre tracts were delineated on PADRE images, speculated that PADRE enhanced the identification of differences in myelin density. Under the hypothesis that the PADRE technique is highly sensitive to the myelin concentration and that it presents MS lesions in terms of myelin loss (demyelination), we evaluated its usefulness for the detection of GM involvement of the juxtacortical lesions of MS patients.

## Materials and Methods

### Patient selection

Our institutional review board (The Hospital Ethics Committee of Medicine and Medical Care, University of Occupational and Environmental Health, Japan) approved this retrospective study and waived informed consent. The patient records/information was anonymized and de-identified prior to analysis.

Thirteen patients (4 men, 9 women, mean age 35.6 years, range 23–52 years) with clinically definite MS^13^ were included. Their median expanded disability status scale score (EDSS) was 4.0 and the mean disease duration was 75.0 months. MS was diagnosed by two neurologists (K.O. with 24 years of experience and H.A. with 25 years of experience in neurology). All patients were clinically evaluated between June 2012 and April 2013 and underwent PADRE in addition to routine brain MRI including T_2_WI, 2D and 3D FLAIR imaging.

### MR imaging

All studies were performed on a 3T MRI system Signa EXCITE 3.0T (GE Healthcare Waukesha, Wisconsin, USA) using a dedicated eight-channel phased-array coil (USA Instruments Aurora, Ohio, USA). PADRE images were obtained with a 3D multi-echo spoiled gradient echo (GRE) sequence.^14^ The imaging parameters were: axial planes covering the brain; number of echo times, 11; first echo time, 4.5 msec; uniform echo time spacing, 5 msec; repetition time, 58.4 msec; flip angle, 15°; band width per pixel, ± 62.5 Hz; field of view, 22 × 16.5 cm; acquisition matrices, 320 × 416; slice thickness, 1.5 mm; imaging time, 7 min 1 sec. A parallel imaging method, the array spatial sensitivity encoding technique, was used with a reduction factor of 2.

All patients were also scanned using our standard brain MRI protocol for MS patients including axial T_2_WI, and 2D and 3D FLAIR imaging. The imaging parameters were 4500/85/1/10.9/512 × 512/2 min 10 sec (repetition time msec/echo time msec/number of excitation/echo-spacing/matrix/imaging time) for T_2_-weighted fast spin-echo (FSE) imaging, and 12,000/140/2600/2/9.1/224 × 256/3 min 20 sec (repetition time msec/echo time msec/inversion time/number of excitation (NEX)/echo-spacing/matrix/imaging time) for 2D FLAIR imaging. T_2_WI and 2D FLAIR images were acquired with a section thickness of 4 mm, an intersection gap of 1 mm, and a field of view of 22 cm. The parallel imaging technique (reduction factor, 2) was used only for T_2_WI. For 3D FLAIR imaging, the parameters were 10,000/141/2478/1/62.5/224 × 224/22 cm/1.2 mm/2.86/5 min 30 sec (repetition time msec/echo time msec/inversion time/NEX/echo train length/matrix/field of view/section thickness/reduction factor/imaging time).

### PADRE technique

PADRE was as described by Kakeda et al.^10^ Its power lies in its phase-difference selection which enhances the magnetic properties of the target tissue. While susceptibility-weighted imaging (SWI) identifies only phase differences related to vessels, especially veins, the PADRE technique classifies and selects various phase differences, *Δθ*, to enhance different tissues. It enhances all tissues on the magnitude image |*ρ|* with the enhancing function *w* (*Δθ*). Finally, the PADRE image *ρ_PADRE_* is reconstructed as:

$$\rho_{\mathrm{PADRE}}=w(\Delta \theta )|\rho |$$

We optimized the reconstitution parameters of PADRE based on Kakeda et al.^10^ and we reconstructed SWI-like images from the MR data.

### Image interpretation

#### Evaluation of artifacts on PADRE images:

To determine the possible rate of artifacts misinterpreted as lesions in the juxtacortical area, PADRE images of five healthy subjects with normal findings on conventional MRI scans were read by two experienced neuroradiologists (J.M. and S.K., with 11 and 16 years of experience in neuroradiology, respectively). Five healthy control subjects (1 man and 4 women) had a mean age of 35.4 ± 8.96 years (range 26–49).

#### Lesion identification:

We defined MS lesions as hyperintense lesions on conventional MR images (T_2_WI, 2D and 3D FLAIR images). On PADRE images, MS lesions were defined as sharply demarcated areas of high signal intensity compared to the adjacent normal-appearing brain parenchyma. One radiologist (K.F. with 6 years of experience in neuroradiology) reviewed the conventional MR images of the 13 MS patients and selected 48 juxtacortical lesions in 6 patients (6 women; mean age, 36 years; range, 23–48 years).The other seven patients had no juxtacortical lesions.

#### Observer study:

**Fig. 1.**
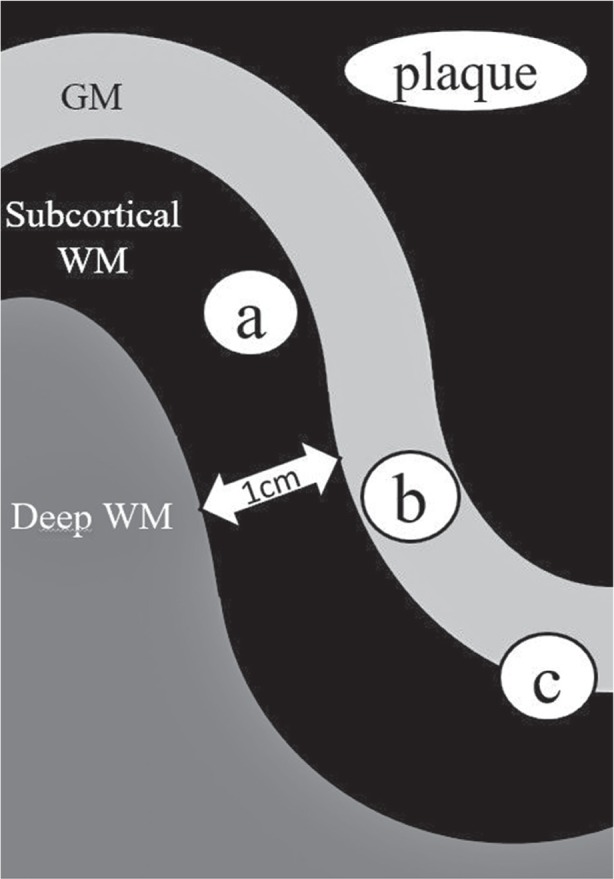
Classification of the 48 juxtacortical MS lesions. Involvement is of (**a**) subcortical WM alone, (**b**) GM alone, (**c**) subcortical WM and GM. We defined the subcortical WM as a WM within a distance of 10 mm from inner edge of the GM. GM, gray matter; MS, multiple sclerosis; WM, white matter.

Two radiologists (J.M. and S.K.) participated in the observer performance study of 48 juxtacortical lesions. At the first reading session they were shown only the conventional MR images. They classified the lesions into three patterns according to their anatomical locations: (a) subcortical WM lesions involving the subcortical WM alone; (b) intracortical (IC) lesions involving the GM alone; (c) mixed GM/subcortical WM (Mixed) lesions involving the both subcortical WM and GM.^15^ We defined the subcortical WM as a WM within a distance of 10 mm from inner edge of the GM. For the analyses, we excluded the white matter lesions further than 10 mm from inner edge of the GM (Fig. 1). At the second reading session MRI and PADRE images were available and the radiologists re-evaluated their prior classification of the juxtacortical lesions. We then compared the number of lesions assigned to each category in the two sessions.

### Statistical analysis

Inter-observer agreement for quantitative and qualitative assessments was calculated as a weighted κ value. The strength of the agreement was considered fair for κ = 0.21–0.40, as moderate for κ = 0.41–0.60, as good for κ = 0.61–0.80, and as excellent for κ = 0.81 or greater.

## Results

### Evaluation of artifacts on PADRE images

**Fig. 2.**
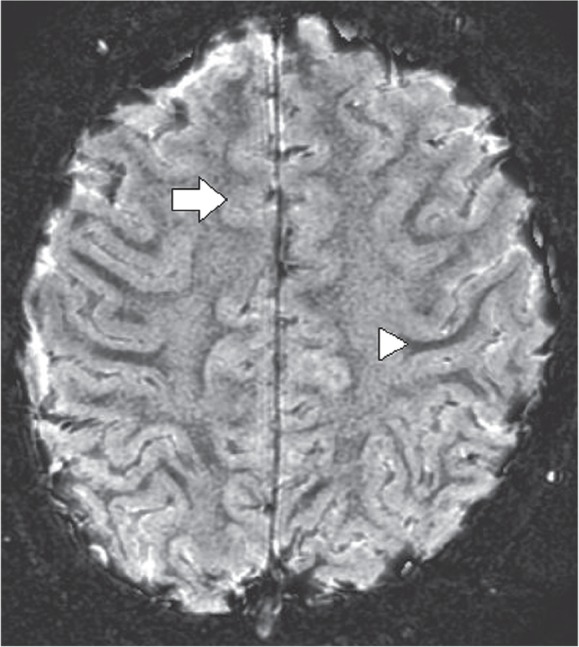
PADRE image of a 35-year-old healthy woman. The signal intensity of the GM is homogeneously slightly hyperintese (arrow). The subcortical WM is homogeneously hypointense vis-à-vis the GM (arrowhead). GM, gray matter; WM, white matter.

Both radiologists agreed that the signal intensity of the GM was homogeneously slightly hyperintense, and that the signal intensity of the subcortical WM was homogeneously hypointense vis-à-vis the GM in all healthy volunteers (Fig. 2). They detected no abnormalities in the juxtacortical area of the volunteers.

### Observer study

**Fig. 3.**
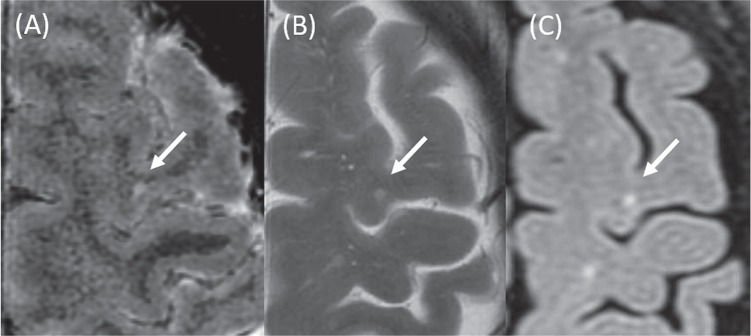
A juxtacortical multiple sclerosis lesion visualized on a PADRE (**A**), a T_2_-weighted imaging (**B**), and a 3D FLAIR image (**C**). The PADRE image shows a lesion involving both the gray matter and U-fiber. On the other images, involvement of the U-fiber only was recognized (arrows). 3D FLAIR, three-dimensional fluid-attenuated inversion recovery; PADRE, phase difference enhanced imaging.

Table 1 shows the results of the observer study for each reading session. Of the 48 juxtacortical lesions, 21 (44%) were judged to involve the GM in the first session. The classification of 15 lesions changed in the second session; all had been judged not to involve the GM. Consequently, a total of 36 lesions (75%) were found to involve the GM on PADRE images (Fig. 3). Interobserver agreement was moderate (kappa = 0.84) for the first- and excellent (kappa = 0.95) for the second reading session.

## Discussion

As our study assessed the potential additional diagnostic value of PADRE for the detection of juxtacortical lesions with GM involvement in MS patients, we evaluated only the lesions that could be detected on conventional MRI images. We found that on PADRE images the number of juxtacortical lesions with GM involvement was greater than on conventional MRI scans and that interobserver agreement was better for PADRE images.

Phase images from GRE sequences depict brain structures at much greater detail than do the corresponding conventional MRI scans. While the origin of the susceptibility contrast on the phase images is not fully understood, differences in the myelin level,^16^ the relative volume and oxygenation state of blood,^17,18^ iron deposition,^16,19,20^ the chemical exchange between water and macromolecular protons,^21^ and the orientation of underlying WM fiber with respect to the main magnetic field^22^ have been suggested. Kakeda et al.^23^ identified nerve fiber as low signal intensity bands on PADRE images; they speculated that the myelin concentration was the main factor that determined the low signal intensity on PADRE images. Based on an earlier study, we attempted to optimize the reconstitution parameters of PADRE to enhance the myelin density.^12^

The subcortical WM fills the space between the deep WM and the GM; it contains a larger amount of nerve fibers, not only the large axonal bundles but also the U-fiber. Our observation that PADRE delineated the subcortical WM as a low signal intensity area suggests a higher myelin content. In contrast, the GM was slightly hyperintense. Although the precise mechanisms underlying the signal intensity of the GM are unclear, the myelin content,^17^ cellular composition, vascularization, and the iron content might affect the signal intensity of the GM on PADRE images. Consequently, the GM and WM are clearly delineated on PADRE images due to differences in the myelin concentration.

The good results we obtained with the PADRE technique may be attributable to spatial resolution differences and to well-defined contrast differences between MS lesions and the surrounding gray and white matter. The spatial resolution for the PADRE sequence is 0.69 × 0.40 × 1.5 mm; for T_2_WI, 2D FLAIR-, and 3D FLAIR imaging it is approximately 0.43 × 0.43 × 4.0 mm, 0.98 × 0.86 × 4.0 mm, and 0.98 × 0.98 × 1.2 mm, respectively. The higher spatial resolution of the PADRE technique may also explain the excellent interobserver agreement we recorded for the depiction of juxtacortical lesions with GM involvement. On PADRE images almost all MS lesions exhibited high signal intensity due to myelin loss within the MS lesions. Therefore, the signal intensity of the juxtacortical lesions, the subcortical WM, and the GM was high-, low-, and slightly high, respectively, yielding well-defined contrast differences between the MS lesions and the subcortical WM and GM.

Involvement of the GM by the juxtacortical lesions is typically not seen on conventional MR images because the lesions are relatively small, manifest poor contrast against the surrounding normal GM, and can be obscured by partial volume effects from cerebral spinal fluid.^24^ It has been reported that the SNR was improved on 3D FLAIR images and that the contrast-to-noise ratio was better than on 2D FLAIR images,^25^ however, it remained difficult to distinguish between juxtacortical lesions with- and without GM involvement. With respect to infratentorial lesions with slightly prolonged T_2_ relaxation times, the DIR technique has advantages over FLAIR imaging.^26–28^ As, besides demyelination, the pathologic features of MS lesions include perivascular cellular infiltration, DIR images are particularly sensitive to these water-rich conditions. PADRE may be less sensitive for depicting these conditions. Consequently, DIR and PADRE images yield different and complementary information for the assessment of MS lesions.

Our study has some limitations. As we have no histologic confirmation (reference standard) for the diagnosis of all MS lesions we evaluated, the juxtacortical lesions we studied may include artifacts. However, since no juxtacortical lesions were found on PADRE images of our healthy subjects, is unlikely that artifacts were misinterpreted as MS lesions. We also did not evaluate intracortical lesions depicted only on PADRE images because there is no consensus reference standard for establishing the diagnosis of MS lesions on such images. We did not specifically focus on correlating our lesion count with clinical and neuropsychologic parameters. Future studies must focus on correlating a well-defined load of GM- and subcortical WM lesions with specific degrees of disability and neuropsychologic function in a large MS patient cohort. Finally, in this study we did not directly compare 3D DIR- and PADRE images.

## Conclusion

Our preliminary results suggest that PADRE and conventional MRI provide complementary information that may be useful for a better understanding of the pathologic processes underlying the manifestation of MS lesions. Our findings suggest that PADRE is useful for detecting GM involvement of the juxtacortical lesions of MS patients.

## Figures and Tables

**Table 1.** The results of the observer study for each reading session

Classification	The first reading session (− PADRE)	The second reading session (+ PADRE)
(a) Subcortical WM lesions	27 (56.3%)	12 (25%)
(b) Intracortical lesions	1 (2.0%)	1 (2.0%)
(c) Mixed GM/subcortical WM lesions	20 (41.7%)	35 (72.9%)

GM, gray matter; PADRE, phase difference enhanced imaging; WM, white matter.
